# Antibiotic Susceptibility and Minimum Inhibitory Concentration for *Stenotrophomonas maltophilia* Ocular Infections

**DOI:** 10.3390/antibiotics11111457

**Published:** 2022-10-22

**Authors:** Margaret Ming-Chih Ho, Ming-Hui Sun, Wei-Chi Wu, Chi-Chun Lai, Lung-Kun Yeh, Yih-Shiou Hwang, Ching-Hsi Hsiao, Kuan-Jen Chen

**Affiliations:** 1 Department of Ophthalmology, Chang Gung Memorial Hospital, Taoyuan 333, Taiwan; 2 College of Medicine, Chang Gung University, Taoyuan 333, Taiwan; 3 Department of Ophthalmology, Chang Gung Memorial Hospital, Keelung 204, Taiwan

**Keywords:** antibiotic susceptibility, endophthalmitis, keratitis, minimum inhibitory concentration, *Stenotrophomonas maltophilia*

## Abstract

*Stenotrophomonas maltophilia* (*S. maltophilia*) is a Gram-negative, opportunistic pathogen that can lead to ocular infections, such as keratitis and endophthalmitis. The purpose of this study was to determine the antibiotic susceptibility and minimum inhibitory concentrations (MICs) of *S. maltophilia* isolates from ocular infections and to evaluate the differences in antibiotic MICs between keratitis and endophthalmitis isolates. The disc diffusion method revealed that *S. maltophilia* isolates exhibited 91% susceptibility to levofloxacin and moxifloxacin and 61% susceptibility to trimethoprim–sulfamethoxazole (TMP–SMX). The E-test indicated that *S. maltophilia* isolates exhibited 40%, 100%, 72%, 91%, 91%, and 93% susceptibility to ceftazidime, tigecycline, TMP–SMX, levofloxacin, gatifloxacin, and moxifloxacin, respectively. The MIC_90_ values of amikacin, ceftazidime, cefuroxime, tigecycline, TMP–SMX, levofloxacin, gatifloxacin, and moxifloxacin were >256, >256, >256, 3, >32, 1, 2, and 0.75 µg/mL, respectively. The geometric mean MICs of ceftazidime, TMP–SMX, levofloxacin, gatifloxacin, and moxifloxacin were significantly lower for the keratitis isolates than for the endophthalmitis isolates (*p* = 0.0047, 0.003, 0.0029, 0.0003, and 0.0004, respectively). Fluoroquinolones showed higher susceptibility and lower MICs for the *S. maltophilia* isolates when compared with other antibiotics. Fluoroquinolones can be recommended for treating *S. maltophilia* ocular infections. Tigecycline and TMP–SMX could be alternative antibiotics for *S. maltophilia* ocular infections.

## 1. Introduction

*Stenotrophomonas maltophilia* is a Gram-negative, opportunistic, nosocomial pathogen that can lead to severe systemic diseases, such as bacteremia, pneumonia, soft-tissue infections, and endocarditis, especially among immunocompromised patients [1,2,3,4]. In ocular infections, *S. maltophilia* could cause keratitis, conjunctivitis, dacryocystitis, scleritis, and endophthalmitis, which may lead to ocular discomfort and visual impairment [5,6,7]. In Taiwan, the most commonly used antibiotics for vision-threatening ocular infections are topical 0.5% levofloxacin for bacterial keratitis and intravitreal ceftazidime (2.25 mg/0.1 mL) and vancomycin (1 mg/0.1 mL) for bacterial endophthalmitis. The treatment of ocular *S. maltophilia* infection has been challenging for ophthalmologists because the *S. maltophilia* strain is resistant to multiple antibiotics, including penicillins, third-generation cephalosporins, aminoglycosides, and imipenem [6,8,9,10] and has variable susceptibility to fluoroquinolones, tigecycline, and trimethoprim–sulfamethoxazole (TMP–SMX) [11,12,13]. One of the most widely discussed antimicrobial resistance mechanisms (ARMs) of *S. maltophilia* was the multidrug-resistance (MDR) efflux pumps [14]. The ATP-binding cassette efflux pump (SmrA), one of the MDR efflux pumps, may contribute to the resistance of fluoroquinolones and tetracycline. Other efflux pumps, such as SmeDEF, one of the most well-known efflux pumps in the resistance nodulation division (RND) family, may also affect the susceptibility to antimicrobials, such as fluoroquinolones, tetracycline, and TMP-SMX. Other ARMs of *S. maltophilia* include β-Lactamases, aminoglycoside-inactivating enzymes, RNase G inactivation, the class I integrons, and the biocide resistance [15,16].

Minimum inhibitory concentrations (MICs) are defined as the lowest concentrations of antimicrobials that will inhibit the visible growth of a microorganism after overnight incubation [17]. It was used to determine the susceptibility of antimicrobials and found the potential treatment for microorganisms. As far as we were aware, reports on the antibiotic MICs for *S. maltophilia* isolates collected from other ocular infections are rare, and whether the MICs for such isolates are similar to those for endophthalmitis isolates is unclear [18,19]. Accordingly, the aim of the present study was to investigate the antibiotic susceptibility—analyzed using MICs—of *S. maltophilia* isolates collected from patients with ocular infections over a 10-year period. 

## 2. Materials and Methods

The study design conformed to the principles of the Declaration of Helsinki and was approved by the Institutional Review Board of Chang Gung Memorial Hospital (IRB number: 202000181B0, 2020/02/06v1), Taoyuan, Taiwan. *S. maltophilia* isolates were retrospectively collected from patients with ocular infections from 1 January 2010 to 30 April 2019. For keratitis, the isolates were collected through corneal scraping; for endophthalmitis, the isolates were collected from anterior chamber or vitreous samples obtained through tapping or vitrectomy.

### 2.1. Antibiotic Susceptibility Testing

All microbiology investigations were performed at the microbiology department of Chang Gung Memorial Hospital, Taoyuan, Taiwan. Different methods were used to identify bacterial culture isolates. From January 2010 to December 2013, conventional microbiological methods were used to identify culture isolates, while from January 2014 to April 2019, matrix-assisted laser desorption/ionization time-of-flight mass spectrometry (MALDI-TOF-MS) was used to identify culture isolates. Conventional microbiological methods, including Gram-staining and biochemical tests, were also employed. In MALDI-TOF-MS, the spectra of the bacteria were automatically measured and compared to reference spectra using an Ultraflextreme mass spectrometer and MALDI-Biotyper 3.0 software (Bruker Daltonics, Karlsruhe, Germany). The reliability of identification in the MALDI Biotyper system was expressed in points. A log (score) of ≥2.0 indicated identification to the species level. The susceptibility of the isolates to various antibiotics was tested using the Kirby–Bauer disc diffusion method on Mueller Hinton blood agar. The Clinical and Laboratory Standards Institute (CLSI, Wayne, PA, USA, 2021) standards [16] were used for interpretation and quality control for each corresponding year. The antibiotics selected for susceptibility testing were levofloxacin, moxifloxacin, and TMP–SMX.

The antibiotics selected for MIC testing were tigecycline, cefuroxime, levofloxacin, TMP–SMX, gatifloxacin, moxifloxacin, amikacin, and ceftazidime. The MICs were determined using susceptibility strips (Epsilometer test (E-test); bioMérieux S.A., Marcy l’Etoile, France). The culture was conducting by taking the cryotubes from the freezer (−80 °C) and scraping one loop of solution with an inoculation ring on the blood agar plate (Trypticase^®^ Soy Agar with 5% Sheep Blood, Nippon Becton Dickinson Co., Ltd., Tokyo, Japan). Then, the plate was incubated at 37 °C for 16 to 18 h. After the incubation, a single colony was selected for subculture. 

The E-test was performed using Mueller Hinton Agar (MHA; CMP^®^, New Taipei City, Taiwan) with the following procedure:

- A suspension from a single colony of an overnight growth blood agar plate was prepared.
- The turbidity of inoculum was adjusted to match 0.5 McFarland standard and was inoculated on MHA within 15 min.
- A sterile cotton swab was dipped into the solution and was rotated inside the tube to remove excess liquid.
- The swab was inoculated over the entire surface of the plate.
- The E-test strip was applied to the plate within 15 min, and the whole plate was put in the incubator within 15 min.

The antibiotic susceptibility of each isolate was determined by comparing the MIC of each agent with the MIC breakpoints according to the CLSI guidelines [20].

### 2.2. Statistical Analysis

Descriptive statistics were used to evaluate the MIC data in this study. MIC_50_, representing the concentration of antibiotics that would inhibit the growth of 50% of isolates, and MIC_90_, representing the concentration of antibiotics that would inhibit the growth of 90% of isolates, were used for the evaluation. The mode was defined as the greatest frequency of MIC distribution for each antibiotic.

Since the MIC data for all antibiotic agents did not follow a normal distribution, the Mann–Whitney *U* test was used to compare the geometric mean (GM) of the MICs (hereafter denoted as GM-MICs) for keratitis isolates with those for endophthalmitis isolates. If the MIC values were more than 256, it was presumed to be 256 for the calculation of GM-MIC; if the MIC values were more than 32, it was presumed to be 32 for the calculation of GM-MIC. The significance level was set at *p* = 0.05. All statistical analyses were performed using MedCalc Statistical Software version 20.009 (MedCalc Software Ltd., Ostend, Belgium, 2021).

## 3. Results

A total of 43 *S. maltophilia* isolates were included in this study. Among our study group, thirty-five isolates were collected from patients with keratitis, and eight were collected from patients with endophthalmitis. The MICs of the antibiotics for *S. maltophilia* are listed in Table 1. Relatively low MIC_50_ values were observed for tigecycline (1 µg/mL), TMP–SMX (0.19 µg/mL), levofloxacin (0.5 µg/mL), gatifloxacin (0.38 µg/mL), and moxifloxacin (0.125 µg/mL). Moreover, relatively low MIC_90_ values were observed for tigecycline (3 µg/mL), levofloxacin (1 µg/mL), gatifloxacin (2 µg/mL), and moxifloxacin (0.75 µg/mL). Accordingly, based on the CLSI guidelines, *S. maltophilia* was determined to exhibit 100% susceptibility to tigecycline, 72% susceptibility to TMP–SMX, 91% susceptibility to levofloxacin and gatifloxacin, 93% susceptibility to moxifloxacin, and 40% susceptibility to ceftazidime. 

Table 2 presents a comparison of the antibiotic MIC_50_, MIC_90_, and GM-MIC values between keratitis isolates and endophthalmitis isolates. The results revealed that the GM-MIC values of ceftazidime, TMP–SMX, levofloxacin, gatifloxacin, and moxifloxacin were significantly lower for the keratitis isolates than for the endophthalmitis isolates (*p* = 0.0047, 0.003, 0.0029, 0.0003, and 0.0004, respectively).

**Figure 1:**
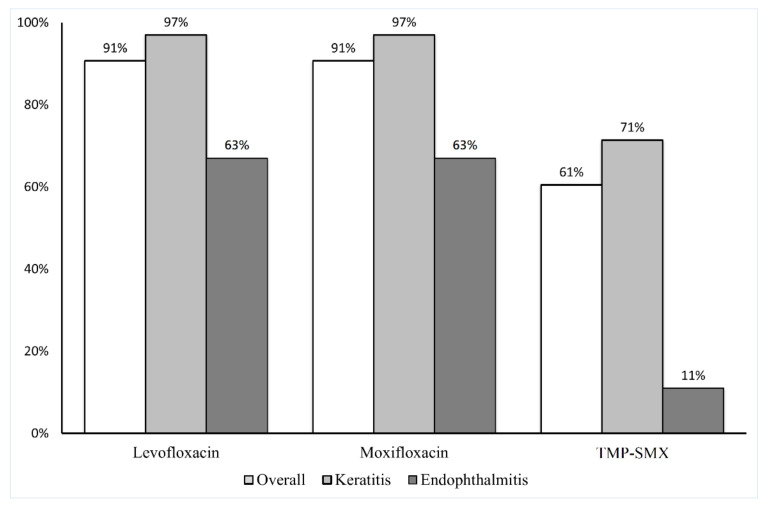
Susceptibility of *S. maltophilia* isolates determined through the Kirby–Bauer disc diffusion method. Of the 43 isolates, 39 (91%) were susceptible to levofloxacin and moxifloxacin, and 26 (61%) were susceptible to TMP–SMX.

**Figure 2:**
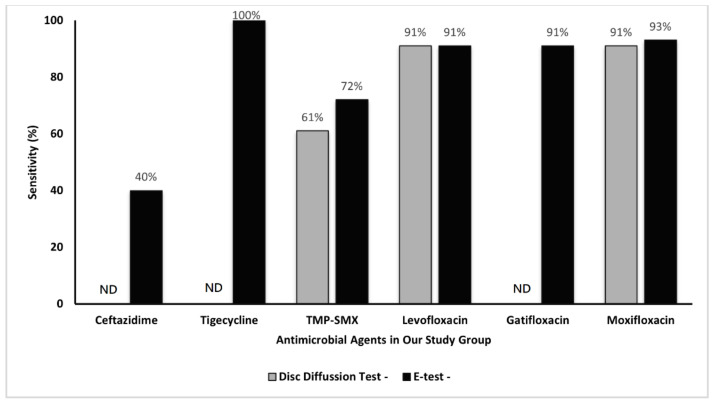
The comparisons of the susceptibility of *S. maltophilia* isolates for each antibiotic using the disc diffusion method and E-test. ND, no data.

Figure 1 demonstrates the susceptibility of the isolates as determined through the Kirby–Bauer disc diffusion method. Of the 43 isolates, 39 (91%) were susceptible to levofloxacin and moxifloxacin and 26 (61%) were susceptible to TMP–SMX. Among the isolates collected from patients with keratitis, 34 (97%) were susceptible to levofloxacin and moxifloxacin and 25 (71%) were susceptible to TMP–SMX. Among the isolates collected from the patients with endophthalmitis, five (63%) were susceptible to both levofloxacin and moxifloxacin, and one (11%) was susceptible to TMP–SMX. The comparisons of the susceptibility of *S. maltophilia* isolates for each antibiotics using the disc diffusion method and E-test are presented in Figure 2.

## 4. Discussion

This study demonstrated the antibiotic susceptibility determined using MICs of *S. maltophilia* isolates obtained from patients with ocular infections. The *S. maltophilia* isolates exhibited a higher susceptibility and lower MICs to fluoroquinolones than to other antibiotics. By comparing the MICs of the antibiotics for *S. maltophilia* isolates collected from various ocular sites, we observed that the GM-MICs of fluoroquinolones, TMP–SMX, and ceftazidime were significantly lower for keratitis isolates than for endophthalmitis isolates. To the best of our knowledge, this study is the first to determine the MICs of antibiotics for *S. maltophilia* keratitis and to compare antibiotic susceptibility between keratitis isolates and endophthalmitis isolates.

Several methods are available for testing the susceptibility of *S. maltophilia* isolates to antibiotic agents [21,22]. The Kirby–Bauer disc diffusion method is among the most commonly used tests for determining antibiotic susceptibility in clinical laboratory settings [23]. This method provides adequate results and is simple and reproducible without the need for any expensive equipment [24]. The E-test is another method for testing the susceptibility of *S. maltophilia* isolates to antimicrobial agents. This test is less labor intensive and more convenient for daily laboratory routines [21]. Yao et al. [21] compared the performance of the agar dilution method and the E-test in determining *S. maltophilia* antibiotic susceptibility and reported that the E-test exhibited an overall agreement of 94% with the agar dilution method. Gülmez et al. [22] compared the performance of the E-test, Phoenix system, and disc diffusion method and discovered that the E-test exhibited acceptable agreement with the Phoenix system and disc diffusion method regarding susceptibility to TMP–SMX and tigecycline. Furthermore, Nicodemo et al. [25] compared the disc diffusion method, E-test, and agar dilution method and discovered that they exhibited good agreement in determining susceptibility to gatifloxacin, TMP–SMX, and tigecycline. Khan et al. [26] also reported similar results for TMP–SMX and levofloxacin, indicating that both were the most active antimicrobials agents for *S. maltophilia* isolates. In the present study, the E-test and disc diffusion method yielded similar susceptibility levels to levofloxacin and moxifloxacin, but the disc diffusion method revealed a lower susceptibility level to TMP–SMX than did the E-test. 

According to the worldwide SENTRY program (1997–2016) which obtained 6467 *S. maltophilia* isolates from different body sites, the *S. maltophilia* isolates were 81.5% and 80.9% susceptible to levofloxacin worldwide and in the Asia-Pacific area based on the CLSI guideline, respectively [27]. Previous studies have reported that *S. maltophilia* isolates obtained from patients with ocular infections exhibited high susceptibility to fluoroquinolones [28,29]. Palioura et al. [28] reported that 96% of isolates collected from 26 patients with infectious keratitis were susceptible to fluoroquinolones. Wu et al. [29] collected *S. maltophilia* isolates from 21 patients with culture-proven keratitis; all collected isolates were susceptible to levofloxacin and moxifloxacin. Our previous study [18] revealed that levofloxacin as well as moxifloxacin were susceptible to *S. maltophilia* in 67% of isolates, and the MIC_90_ values were lowest for the *S. maltophilia* isolates in comparison with other antimicrobial agents (1, 8, and 12 µg/mL for levofloxacin, moxifloxacin, and gatifloxacin, respectively) from isolates from endophthalmitis. In the present study, the fluoroquinolones were over 90% susceptible for *S. maltophilia* isolates in both the disk diffusion test and the E-test. These results were consistent with previous studies and showed that the fluoroquinolones had presented high in vitro activity for *S. maltophilia* isolates. The possible explanation for the decrease in MICs for *S. maltophilia* isolates is related to the MDR efflux pumps. The SmeDEF, one of the efflux pumps in the MDR efflux system, is related to a two-to-eight-fold decrease of the MIC values of fluoroquinolones [30].

We also discovered that the *S. maltophilia* isolates exhibited 100% susceptibility to tigecycline at a low MIC_90_ value (3 µg/mL), indicating that tigecycline could be a clinical candidate for the treatment of *S. maltophilia* ocular infections. A multicentered study [31], including 1586 *S. maltophilia* isolates collected from the bloodstream and respiratory tract, demonstrated that tigecycline (to which 95.5% of the isolates susceptible at an MIC of ≤2 μg/mL) and TMP–SMX (to which 96.0% of the isolates were susceptible at an MIC of ≤2 μg/mL for TMP and 38 μg/mL for SMX) were the only tested antibiotics associated with more than 94% susceptibility. In a 10-year long multi-center study in Taiwan [32], *S. maltophilia* isolates were most susceptible to minocycline (99.7%) and tigecycline (96%) and then to TMP–SMX (82.5%) and levofloxacin (79.6%); the MIC_90_ values of both minocycline and tigecycline were 1 μg/mL, lower than those of TMP–SMX (8 μg/mL) and levofloxacin (4 μg/mL). The use of contact lenses may constitute a risk factor for *S. maltophilia* keratitis. Watanabe et al. [19] tested 40 *S. maltophilia* strains collected from contact lens cases, contact lenses, and eye swabs of contact lens wearers, including 27 asymptomatic wearers and 13 patients with keratitis; they reported that all isolates in their study were susceptible to tigecycline. In our previous study [29], 7 of 21 patients with *S. maltophilia* keratitis had a history of soft contact lens or therapeutic contact lens use. All isolates collected from these seven patients were susceptible to levofloxacin and moxifloxacin. Isolates from five patients were tested for tigecycline and were determined to be susceptible to tigecycline. Therefore, tigecycline could be a potential therapeutic option for the treatment of *S. maltophilia* ocular infections, but additional clinical evidence is required to confirm its efficacy. 

TMP–SMX exhibited a lower MIC_90_ value for *S. maltophilia* isolates collected from patients with endophthalmitis (12 μg/mL) than for those collected from patients with keratitis (>32 μg/mL); by contrast, it exhibited lower MIC_50_ (0.19 μg/mL) and GM-MIC (0.350 μg/mL) values for isolates collected from patients with keratitis. TMP–SMX has been used to treat *S. maltophilia* systemic infections [33]. For *S. maltophilia* ocular infections, TMP–SMX has been used to reduce the risk of recurrent toxoplasma retinochoroiditis, but it has not been used routinely as a monotherapy [34]. Tatman-Otkun et al. [35] reported that *S. maltophilia* isolates exhibited 98.1% susceptibility to TMP–SMX, and the disc diffusion and E-test results regarding *S. maltophilia* isolate susceptibility to TMP–SMX were consistent. Khan et al. [36] reported that in blood steam isolates, TMP-SMX exhibited a more than 90% susceptibility in MicroScan, broth microdilution, and Phoenix testing using CLSI breakpoints. In the present study, the disc diffusion method revealed that the *S. maltophilia* isolates showed 61% susceptibility to TMP–SMX, especially for isolates collected from the patients with keratitis (71%) but not for those collected from the patients with endophthalmitis (11%). Similar results were obtained through the E-test, which revealed a 72% susceptibility level for the *S. maltophilia* isolates. Our results provide in vitro evidence that TMP–SMX may be effective in treating *S. maltophilia* keratitis.

In our study, amikacin, ceftazidime, and cefuroxime showed relatively high GM-MICs in both the keratitis and endophthalmitis groups. The relatively low susceptibility for these antimicrobials might be explained in several mechanisms. The 6′-N-aminoglycoside acetyltransferase as well as proteases, such as ClpA and HtpX, were related to the aminoglycoside resistance in previous studies [15,37]. On the other hand, the inactivation of L1 metallo-β-lactamases and class A L2 β-lactamases were related to the increase resistance of cephalosporins [30]. Future studies on the mechanism of resistance of amikacin, ceftazidime, and cefuroxime against *S. maltophilia* were needed to broaden the treatment choices of *S. maltophilia* infections.

This study has several limitations. For example, it applied a retrospective design and used different methods for clinical sample collection. For keratitis, isolates were collected through corneal scrapping, whereas for endophthalmitis, isolates were collected from the aqueous humor or vitreous through tapping and vitrectomy. Moreover, the number of endophthalmitis isolates was lower than that of keratitis isolates. Despite these limitations, this study presents in vitro evidence for the antibiotic susceptibility of *S. maltophilia* ocular infections, but further research is warranted to identify the most effective antibiotics against *S. maltophilia* ocular infections. To the best of our knowledge, this study is the first to compare the antibiotic susceptibility of *S. maltophilia* keratitis and endophthalmitis isolates and to determine the MICs of antibiotics for *S. maltophilia* keratitis.

## 5. Conclusions

Treating *S. maltophilia* ocular infections is a clinical challenge because of their resistance to multiple antibiotics. Fluoroquinolones are recommended for *S. maltophilia* ocular infections, especially for keratitis, because they are associated with higher susceptibility at lower MICs compared with other antibiotics. Furthermore, tigecycline and TMP–SMX can be used as alternative treatments for *S. maltophilia* ocular infections. Our findings regarding the antibiotic susceptibility and MICs of the *S. maltophilia* isolates may help ophthalmologists in treating patients with *S. maltophilia* ocular infections

## Figures and Tables

**Table 1:** Minimum Inhibitory Concentrations and Antibiotic Susceptibilities of *Stenotrophomonas maltophilia* Isolates.

Antibiotics	n	MIC (µg/mL)					Susceptibility (%)		
		MIC_50_^a^	MIC_90_^b^	GM MIC	Mode *	MIC Range	S	I	R
Amikacin	43	12	>256	16.732	>256	2–>256	-	-	-
Ceftazidime	43	48	>256	21.562	>256	0.064–>256	40	7	53
Cefuroxime	43	>256	>256	242.288	>256	24–>256	0	2	98
Tigecycline	43	1	3	1.249	1	0.38–8	100	0	0
TMP-SMX	43	0.19	>32	0.668	>32	0.047–>32	72	0	28
Levofloxacin	43	0.5	1	0.575	0.5	0.064–>32	91	7	2
Gatifloxacin	43	0.38	2	0.424	0.38	0.094–12	91	2	7
Moxifloxacin	43	0.125	0.75	0.172	0.125	0.032–8	93	7	2

GM: geometric mean, MIC: minimum inhibitory concentration, ^a^ MIC_50_ values, ^b^ MIC_90_ values, I: intermediate, S: susceptible, TMP-SMX: trimethoprim–sulfamethoxazole, R: resistant; * the value among all observations that occurs at the greatest frequency. -: indicates absence of MIC breakpoint. The MIC breakpoints are cited from the 31st edition of the Clinical and Laboratory Standards Institute (CLSI) M100 guidelines (April, 2021) [20].

**Table 2:** Minimum Inhibitory Concentrations of *S.* *maltophilia* Isolates from Keratitis and Endophthalmitis.

Antibiotics	Keratitis MIC (µg/mL)				Endophthalmitis MIC (µg/mL)				*p* Value
	MIC_50_^a^	MIC_90_^b^	GM MIC ^†^	MIC Range	MIC_50_	MIC_90_	GM MIC ^†^	MIC Range	
Amikacin	16	>256	18.817	2–>256	4	>256	10.212	2–>256	0.0690
Ceftazidime	24	>256	14.931	0.064–>256	>256	>256	174.181	8–>256	0.0047 *
Cefuroxime	>256	>256	239.259	24–>256	>256	>256	256.000	all > 256	0.6121
Tigecycline	1	2	1.127	0.38–4	2	8	2.476	0.5–8	0.0535
TMP-SMX	0.19	>32	0.350	0.047–>32	12	12	5.992	0.5–12	0.0030 *
Levofloxacin	0.5	1	0.447	0.064–1	1	12	1.901	0.5–12	0.0029 *
Gatifloxacin	0.25	0.75	0.326	0.094–3	0.5	12	1.904	0.25–12	0.0003 *
Moxifloxacin	0.125	0.38	0.128	0.032–1.5	0.25	8	0.939	0.125–8	0.0004 *

GM: geometric mean, MIC: minimum inhibitory concentration, ^a^ MIC_50_ values, ^b^ MIC_90_ values, TMP-SMX: trimethoprim–sulfamethoxazole; ^†^ if the MIC value was >256, the MIC was presumed to be 256 for the calculation of GM MIC; if the MIC value was >32, the MIC was presumed to be 32 for the calculation of GM MIC. * *p* < 0.05.

## Data Availability

The data are not publicly available as they contain information that could compromise the privacy of the research participants.
